# O016. Does migraine follow benign paroxysmal torticollis?

**DOI:** 10.1186/1129-2377-16-S1-A143

**Published:** 2015-09-28

**Authors:** Maria Chiara Bernucci, Roberto Frusciante, Alessandro Capuano, Samuela Tarantino, Federico Vigevano, Massimiliano Valeriani

**Affiliations:** Headache Centre, Ospedale Bambino Gesù, IRCCS, Rome, Italy; Center for Sensory-Motor Interaction, Aalborg University, Aalborg, Denmark

## Background

Migraine equivalents are clinical conditions which often involve children who do not complain of headache. They include abdominal migraine, motion sickness, limb pain, cyclical vomiting, benign paroxysmal vertigo, and benign paroxysmal torticollis (BPT). The aim of our study was to investigate whether children referred to us for BPT have developed migraine at a distance from our first observation.

## Methods

Forty-one children were included in the study. Only 36 families could be contacted by phone, but 2 of them refused to answer our questionnaire. Therefore, the present results were obtained from 34 children (22 girls and 12 boys).

## Results

Migraine could be diagnosed in 14 children (41%), while the remaining 20 patients (59%) did not complain of headache. At the moment of our interview, children who had developed migraine had a mean age of 5 years, while the mean age of non-migrainous children was 3.5 years. Among migraine children, 43% developed it when they were 4 years, 21% at the age of 3, and 14% when they were 7 years old. The last three migraineurs developed migraine at the age of 5 years, 13 years and 18 months, respectively. Moreover, 55% of patients had developed other migraine equivalents. In particular, 73% children had abdominal migraine, 55% vertigo, 45% limb pain, 27% motion sickness, 27% cyclical vomiting. As for the paroxysmal torticollis time course, two children have had only one event, one child had been still presenting episodes of torticollis, while in the remaining patients the torticollis events had not occurred for some years.

## Conclusions

Our findings suggest that migraine follows BPT in approximately half of the children within the age of 13 years. Moreover, BPT is often associated to other migraine equivalents. Considered all together, all these periodic syndromes increase the risk of developing migraine.

Written informed consent to publish was obtained from the patient(s).

